# Mesenchymal stem cell-like properties of CD133^+^ glioblastoma initiating cells

**DOI:** 10.18632/oncotarget.9658

**Published:** 2016-05-27

**Authors:** Lorena Favaro Pavon, Tatiana Tais Sibov, Daniela Mara de Oliveira, Luciana C. Marti, Francisco Romero Cabral, Jean Gabriel de Souza, Pamela Boufleur, Suzana M.F. Malheiros, Manuel A. de Paiva Neto, Edgard Ferreira da Cruz, Ana Marisa Chudzinski-Tavassi, Sérgio Cavalheiro

**Affiliations:** ^1^ Department of Neurology and Neurosurgery, Escola Paulista de Medicina-Universidade Federal de São Paulo (EPM-UNIFESP), São Paulo, Brazil; ^2^ Hospital Israelita Albert Einstein (HIAE), Experimental Research, São Paulo, Brazil; ^3^ Department of Genetics and Morphology, Universidade de Brasília, Brasília, Brazil; ^4^ Allergy and Immunopathology Graduate Program, Faculdade de Medicina, Universidade de São Paulo (USP), São Paulo, Brazil; ^5^ Faculdade de Ciências Médicas da São Casa de São Paulo, São Paulo, Brazil; ^6^ Biochemistry and Biophysics Laboratory, Butantan Institute, Neuro-Oncology Program, São Paulo, Brazil; ^7^ Hospital Israelita Albert Einstein (HIAE), Neuro-Oncology Program, São Paulo, Brazil; ^8^ Discipline of Nephrology, Escola Paulista de Medicina-Universidade Federal de São Paulo (EPM-UNIFESP), São Paulo, Brazil

**Keywords:** neurospheres, CD133^+^, MSC immunophenotype, TEM, tumorigenesis in vivo

## Abstract

Glioblastoma is composed of dividing tumor cells, stromal cells and tumor initiating CD133^+^ cells. Recent reports have discussed the origin of the glioblastoma CD133^+^ cells and their function in the tumor microenvironment. The present work sought to investigate the multipotent and mesenchymal properties of primary highly purified human CD133^+^ glioblastoma-initiating cells. To accomplish this aim, we used the following approaches: *i)* generation of tumor subspheres of CD133^+^ selected cells from primary cell cultures of glioblastoma; *ii)* analysis of the expression of pluripotency stem cell markers and mesenchymal stem cell (MSC) markers in the CD133^+^ glioblastoma-initiating cells; *iii)* side-by-side ultrastructural characterization of the CD133^+^ glioblastoma cells, MSC and CD133+ hematopoietic stem cells isolated from human umbilical cord blood (UCB); *iv)* assessment of adipogenic differentiation of CD133^+^ glioblastoma cells to test their MSC-like *in vitro* differentiation ability; and *v)* use of an orthotopic glioblastoma xenograft model in the absence of immune suppression. We found that the CD133^+^ glioblastoma cells expressed both the pluripotency stem cell markers (Nanog, Mush-1 and SSEA-3) and MSC markers. In addition, the CD133^+^ cells were able to differentiate into adipocyte-like cells. Transmission electron microscopy (TEM) demonstrated that the CD133^+^ glioblastoma-initiating cells had ultrastructural features similar to those of undifferentiated MSCs. In addition, when administered *in vivo* to non-immunocompromised animals, the CD133^+^ cells were also able to mimic the phenotype of the original patient's tumor. In summary, we showed that the CD133^+^ glioblastoma cells express molecular signatures of MSCs, neural stem cells and pluripotent stem cells, thus possibly enabling differentiation into both neural and mesodermal cell types.

## INTRODUCTION

Glioblastoma has a polyclonal nature and is composed of dividing tumor cells, tumor initiating cells and stromal cells [1, 2]. Glioblastoma initiating cells, also called cancer stem cells (CSCs), are characterized as a CD133^+^ cell population within a tumor that possess the abilities to self-renew, generate neurospheres *in vitro* and reproduce the original tumor when administered *in vivo* to immunocompromised animals [3, 4, 5, 6, 7]. CD133^+^, a pentaspan membrane glycoprotein, has been used as a biomarker for glioblastoma initiating cells [3, 8, 9, 10, 11].

Recent reports have discussed the origin of the glioblastoma CD133^+^ cells and their functions in the tumor microenvironment [11, 12, 13, 14]. It is believed that glioblastoma CSCs arise through the neoplastic transformation of normal neuronal stem cells, because both cells are phenotypically CD133 positive. However, regulators of stem cell function (pluripotency markers) have also been implicated in cancer pathogenesis [15, 16, 17, 18, 19]. Furthermore, the grade of the malignancy of glioblastoma and the efficiency of neurosphere formation increases according the expression level of Mush-1 [16].

The differentiation potential of glioblastoma CSCs is not restricted to neural lineages, and the CSCs can also differentiate into mesenchymal stem cells (MSCs) [20]. MSCs are multipotent stromal cells that differentiate into mesodermal lineages and have important immunomodulatory functions [21, 22]. MSCs are plastic-adherent under standard culture conditions and differentiate into osteoblasts, adipocytes and chondroblasts *in vitro*. They express CD29, CD105, CD73, CD44 and CD90, and lack expression of CD45, CD34, CD31, CD133, CD14, CD79a and HLA-DR cell surface markers.

Tso and coworkers [23] have observed that the immortalized glioblastoma cell lines (W-98, D-431, F-502 and S-496) express surface antigen profiles similar to those of MSCs, because these cell lines express variable levels of CD44, CD90, CD105 and CD29, and do not express CD14, CD34 and CD31 cell surface markers.

In addition, forced expression of exogenous Nanog in MSCs leads these cells to differentiate into neural cells, even though this pluripotent embryonic marker is not expressed in neural stem cells [24].

It is unclear whether the CD133^+^ glioblastoma-initiating cells are derived from transformed progenitor cells or whether the heterogeneity of glioblastoma cells results in activation of genes that elicit mesenchymal properties and contribute to the formation of the tumor stroma, which is essential for the growth and maintenance of glioblastomas [2, 25]. Secreted factors from all cells present in the tumor mass are able to diffuse through the peritumoral stroma, thereby creating a permissive microenvironment for malignant progression [26, 27]. In addition, the immunosuppressive characteristics of MSCs may protect tumor cells from attack by immune cells.

In the present study, we show that highly purified, neurosphere-forming CD133^+^ cells, obtained from human glioblastomas, express the cell marker profile characteristics of MSCs and the pluripotency markers, SSEA-1, Mush-1 and Nanog. Furthermore, these cells are capable of differentiating into of mesodermal adipogenic-like cells. In addition, when administered *in vivo* to animals in the absence of immune suppression, the CD133^+^ cells are also able to mimic the phenotype of the original patient's tumor, thus confirming that they have characteristics of CSCs.

## RESULTS

### The establishment of tumor subspheres of CD133+ selected cells from primary cell cultures of glioblastomas

**Figure 1:**
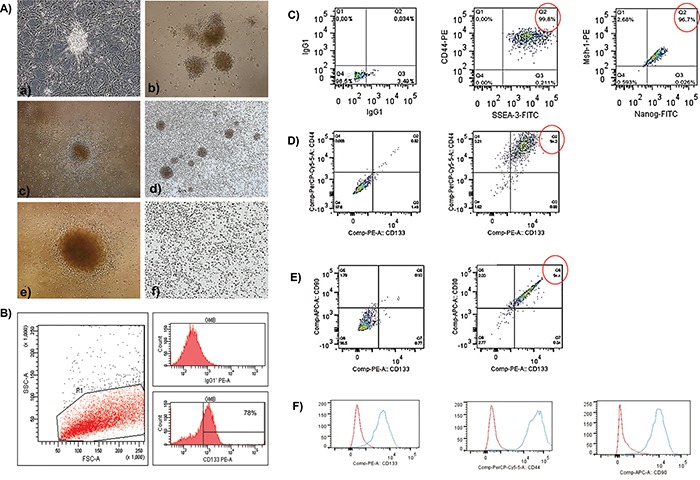
**A, B.** The establishment of human glioblastoma primary cell culture (A-a). Isolation of tumor neurospheres derived from glioblastoma primary cell culture. (A-b, c) Purification of glioblastoma cells from tumor subspheres using CD133 microbeads. Immunophenotypic characterization by using flow cytometry to evaluate the efficiency of magnetic cell separation for the antigenic marker, CD133 (78.0%). (B) CD133^+^ glioblastoma cells were able to further generate subspheres. Culture of glioblastoma subspheres (A-d, e) compared with the absence of subspheres obtained from CD133^−^ fractions. (A-f) Representative figure of five glioblastoma samples at 400X magnification. **C-F.** Immunophenotyping of CD133^+^ glioblastoma cells by using flow cytometry. (C) First plot shows the isotype control. The second and third plots show the staining for CD44 and SSEA-3 (99.8%) and Nanog and MSh-1 (96.7%), respectively. (D) The first plot is an unstained sample, and the second plot shows the staining for CD44 and CD133 (94.0%). (E) The first plot is an unstained sample, and the second plot shows the staining for CD90 and CD133 (94.4%). (F) Red (isotype control); Blue (stained sample).

Primary cell cultures were generated from glioblastoma mass samples (Figure 1A-a). These cells were homogenous, displayed fusiform format and were arranged in multidirectional bundles in culture (Figure 1A-a). Robust neurospheres were generated *in vitro* after glioblastoma cell dissociation (Figure 1A-b, c). As expected, glioblastoma neurospheres selected by using a CD133^+^ affinity column showed a higher content of CD133 positive cells (78%) (Figure 1B). After the dissociation of the neurospheres, the CD133^+^ cells were able to further generate subspheres with well-defined morphology (Figure 1A-d, e), whereas the negative fraction (the CD133^−^ cells) was unable to generate subspheres (Figure 1A-f).

### Immunophenotyping of the CD133^+^ glioblastoma cells by using flow cytometry

Flow cytometry analyses showed that the CD133^+^ cells highly expressed CD44 (94.0%) and CD90 (94.4%) (Figure 1D, 1E). In addition, a percentage of these cells also co-expressed CD44 and SSEA-3 (99.8%), as well as Mush-1 and Nanog (96.7%) (Figure 1C).

**Figure 2:**
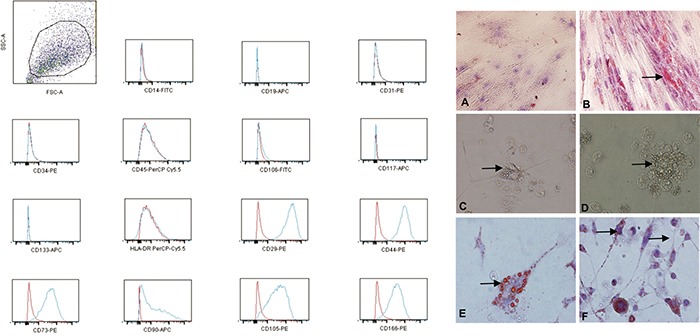
The increased expression of the mesenchymal markers (CD29, CD44, CD73, CD90, CD105 and CD166) and low or no expression of the MHC class I antigens, HLA-DR and the hematopoietic/vascular cells markers (CD14, CD31, CD34, CD45 and CD106) on the CD133+ glioblastoma cells; red: isotype control; blue: stained sample **A-F.** The adipogenic differentiation of the MSCs and glioblastoma CD133^+^ cells as detected by the formation of intra-cytoplasmic lipid droplets stained with Oil Red O (black arrow). (A) CD133^−^ (control). (B) Adipogenic differentiation of the MSCs. (C-F) The adipogenic differentiation of the CD133^+^ glioblastoma cells. (A, B) Magnification: 200X. (C-F) Magnification: 600X.

FACS analysis showed that the glioblastoma CD133^+^ cells expressed the typical mesenchymal markers CD29, CD44 (hyaluronic receptor), CD73, CD90, CD105 (endoglin) and CD166. In addition, our analysis showed that, similarly to MSCs, the CD133^+^ cells did not express high levels of either HLA-DR or the hematopoietic and vascular cell markers CD14, CD31, CD34, CD45 and CD106 (Figure 2).

### Adipogenic differentiation of the CD133^+^ glioblastoma cells

We confirmed that the glioblastoma CD133^+^ adherent cells differentiated into adipocyte-like cells after 21 d by using Oil Red O staining (Figure 2E, 2F). Compared with the CD133^−^ control cells (Figure 2A), these cells showed morphological changes, including a fusiform or fibroblastic morphology and peripheral basophilic nuclei due to the presence of many lipid droplets (Figure 2C–2F). Oil Red O staining was used to detect adipocyte-like UC-MSCs as a positive control (Figure 2B).

### Ultrastructural characterization of the CD133^+^ hematopoietic stem cells (UCBs) and glioblastoma cells

**Figure 3:**
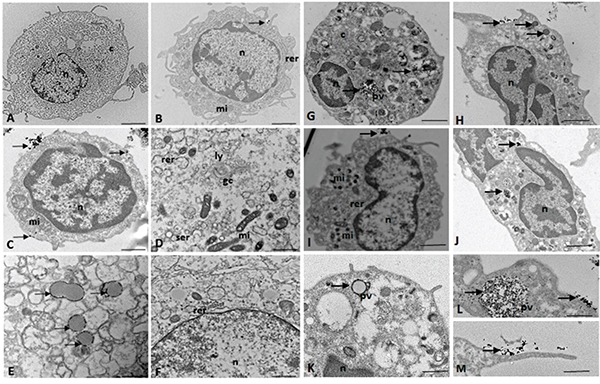
(A-F) TEM of the CD133^+^ hematopoietic stem cells (UCB) n = nucleus, c = cytoplasm, mi = mitochondria, rer = rough endoplasmic reticulum, ser = smooth endoplasmic reticulum, pv = pinocytic vesicles, li = lipid droplets, gc = Golgi complex, ly = lysosomes, arrow = electron-dense granules or magnetic beads. TEM of the CD133^+^ glioblastoma stem cells. n = nucleus, c = cytoplasm, mi = mitochondria, rer = rough endoplasmic reticulum, pv = pinocytic vesicles, arrow = electron-dense granules or magnetic beads. Scale: **A-C, G-J.** 5.0 μm; **F, K.** 2.0 μm; **D, E.** 1.0 μm; **L, M.** 0.5 μm.

Using electron microscopy for ultrastructural analysis, we observed the presence of electron-dense granules on the cell surface of the CD133^+^ glioblastoma and UCB cells, thus demonstrating the presence of anti-CD133 monoclonal antibodies bound to magnetics beads recognizing the CD133 membrane proteins on the cell surface (Figure 3B, 3C, 3H, 3I, 3L, 3M).

Electron micrographs showed that the CD133^+^ cells had a round morphology (Figure 3A, 3B, 3C, 3G, 3I, 3K), with some discrete cytoplasmic projections (Figure 3A, 3B, 3H, 3M). The nuclei occupied the majority of the cells, and there was little cytoplasm in the CD133^+^ cells (Figure 3A, 3B, 3C, 3H, 3I, 3J). Clear nuclear envelopes and chromatin with numerous heterochromatic granules were also observed (Figure 3B, 3C, 3H, 3J).

In the cytoplasm of the CD133^+^ cells, we observed the presence of numerous mitochondria, whose morphology varied from circular and electron dense to large and elongated with well-defined cristae (Figure 3D, 3I).

TEM detected the presence of smooth and rough endoplasmic reticulum and Golgi apparatus in CD133^+^ hematopoietic stem cells (UCB) (Figure 3B, 3D, 3F).

Electron-dense signals related to magnetic microbeads were observed in the cytoplasm of the CD133^+^ glioblastoma cells, which suggested that endocytosis (pinocytosis) facilitated microbead internalization (Figure 3G, 3L).

Both the UCB and glioblastoma CD133^+^ cells incorporated microbeads through their small cytoplasmic projections (Figure 3M), which resulted in clumps of the particles around the pynocytic vesicles (Figure 3E, 3L).

### Ultrastructural characterization of the UC-MSCs

**Figure 4:**
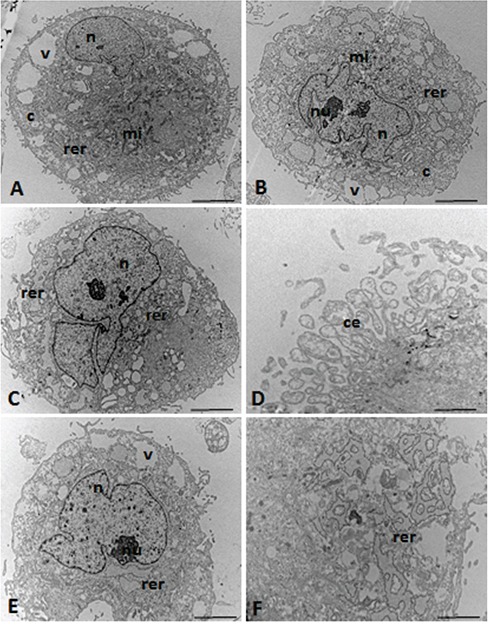
TEM micrographs of the UC-MSCs n = nucleus, nu = nucleolus, c = cytoplasm, mi = mitochondria, rer = rough endoplasmic reticulum, v = vacuoles, ce = cytoplasmic expansions. Scale: **A-C, E.** 5.0 μm; **D, F.** 0.5 μm.

Ultrastructural analysis of the MSCs showed a spindle morphology with cytoplasmic extensions (Figure 4D) and oval nuclei (Figure 4B, 4C, 4E) containing dispersed chromatin and visible nucleoli (Figure 4B, 4C, 4E).

Figure 4B shows the irregular nucleus with a clear nucleolus, thus suggesting high levels of protein synthesis in the nuclear process. The cytoplasm of these cells showed well-developed rough endoplasmic reticulum, and circular and lamellar (Figure 4B, 4C, 4E, 4F) as well as elongated or round mitochondria (Figure 4A, 4B).

### Detection of multimodal iron oxide nanoparticles conjugated to Rhodamine-B (MION-Rh) in the CD133^+^ glioblastoma cells

**Figure 5:**
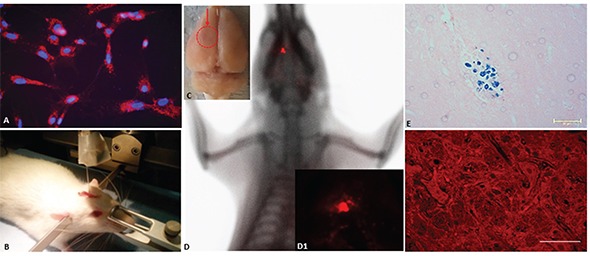
**A.** Fluorescence detection of MION-Rh labeling in the CD133^+^ glioblastoma cells. Magnification: 600X. **B.** Stereotaxic implantation of the MION-Rh labeled CD133^+^ glioblastoma cells labeled in the tumor experimental models. **C.** *Ex vivo* brain imaging; arrow = region of the tumor. **D.** *In vivo* detection of the glioblastoma using combined fluorescence and X-ray tomography. **D1.** Fluorescence image detail. **E.** Immunohistochemical analysis for Prussian blue staining of the tumor. **F.** Fluorescence assay of the tumor. (E) Magnification: 400X. (F) Magnification: 600X.

A qualitative evaluation of the intracellular distribution of MION-Rh in CD133^+^glioblastoma cell was performed by using fluorescence microscopy with a Rh-B filter (530 nm and 550 nm). The presence of red fluorescence showed that MION-Rh was internalized as intracellular granules (Figure 5A).

### The *in vivo* detection of glioblastoma tumor growth by using fluorescence, X-ray and histopathological analysis

Tumors were generated after stereotaxic implantation of glioblastoma CD133^+^ cells labeled with MION-Rh (Figure 5B), and the progression of tumor growth was monitored by using combined fluorescence and X-ray detection (Figure 5C, 5D). On day 28, visible tumors were detected with fluorescence staining (Figure 5D1 and 5F) and Prussian blue staining (Figure 5E).

**Figure 6:**
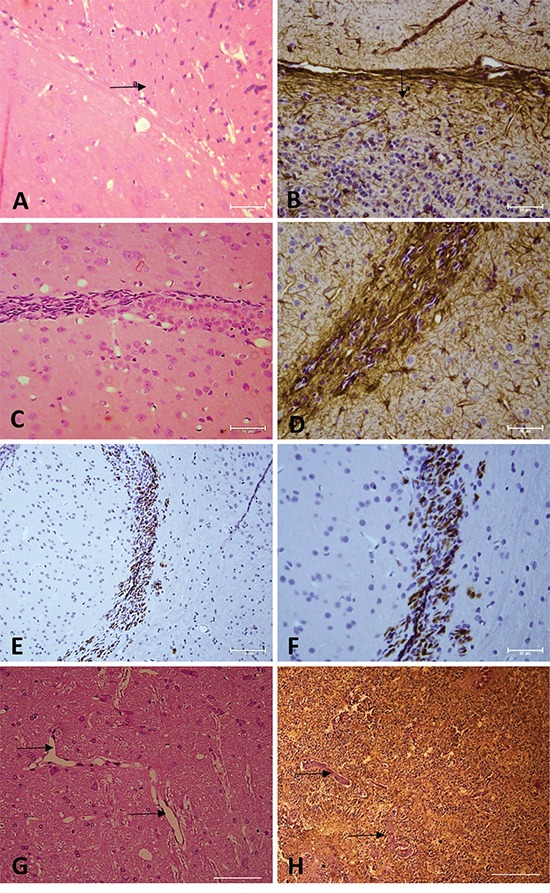
**A, C, G.** Hematoxylin and eosin staining. **B, D, H.** Immunohistochemical analysis for GFAP. **E, F.** Immunohistochemical analysis for Ki67. Arrow = vascular proliferation. Arrow a = tumor area. (A, B, C, E, G, H) Magnification: 400X. (D, F) Magnification: 600X.

Histopathological examination showed that the tumors exhibited high cellularity, nuclear atypia (Figure 6A, 6B, 6C), invasiveness (Figure 6C, 6D, 6E, 6F) and vascular proliferation (Figure 6G, 6H). Immunohistochemical analysis for GFAP confirmed tumor formation originated in the glia (Figure 6B, 6D, 6H), and Ki67 detection revealed a high number of cycling cells in the tumor tissue (Figure 6E, 6F).

## DISCUSSION

The present work demonstrated the mesenchymal stem cell-like properties of CD133+ glioblastoma-initiating cells by examining the pluripotency, stemness characteristics and differentiation potential of CD133^+^ glioblastoma cells.

We described a successful method for isolation of a CD133+ cell population and the establishment of glioblastoma neurospheres from primary cultures. The neurospheres demonstrated a higher concentration of CD133^+^ cells (78%), and the enriched CD133^+^cell fraction was able to further generate clonogenic cells or subspheres with well-defined morphology, thus highlighting the importance of the CD133 marker in the selection and enrichment of these cells from glioblastoma neurospheres.

The immunophenotypic profile of the CD133+ cells showed that these cells expressed high levels of CD44 and CD90 cell-surface glycoproteins. Recently, the CD90 stem cell marker has been identified as a prognostic marker for high-grade gliomas, and CD44 has been shown to be a potential metastatic marker [28, 29]. Bahnassy and colleagues [30] have shown that aberrant expression of cancer stem cell markers (CD133, CD90 and CD44) contributes to tumor progression. In fact, according to our *in vivo* results, the CD133+/CD90+/CD44+ tumor stem cells may be responsible for dissemination of glioblastoma, which leads to reduced patient survival [30]. The glioblastoma CD133^+^ cells also co-express significant levels of SSEA-3 (stage-specific embryonic antigen 3), Mush-1 (neural stem cell marker) and Nanog (transcriptional regulator involved proliferation and self-renewal of embryonic stem (ES) cells). The expression of SSEA-3 and Nanog demonstrate that the CD133^+^ cells are multipotent, whereas expression of Mush-1 indicates their neuronal origin. Therefore, CD133^+^, CD90^+^, SSEA-3, Nanog and Mush-1 may be used as enrichment markers for glioblastoma CSCs [13, 16, 24, 29, 30].

Tso and colleagues [23] have observed that some immortalized glioblastoma cell lines (W-98, D-431, F-502, and S-496) express molecular markers that are associated with MSCs. In addition, these glioblastoma cell lines can be induced to differentiate into multiple mesenchymal lineage-like cell types. In this study, we observed that the CD133^+^ glioblastoma cells expressed molecular markers associated with the MSC phenotype (CD29, CD44, CD73, CD90, CD105 and CD166). Furthermore, the CD133+ cells were also able to differentiate into adipocyte-like cells. These cells had fusiform or fibroblastic morphology and peripheral basophilic nuclei due to the presence of many lipid droplets, characteristics similar to those of MSCs.

To our knowledge, this is the first study to provide the ultrastructural characterization of CD133^+^ cells from two different sources: hematopoietic stem cells (UCB) and glioblastomas. Both the UCB and glioblastoma CD133^+^ cells included anti-CD133 monoclonal antibodies bound to magnetic beads, which were observed as electron-dense granules on the cell surface and were internalized by pynocytic vesicles. The ultrastructural analysis of CD133^+^ hematopoietic stem cells [31] served as a control for the highly purified glioblastoma CD133^+^ cells from the primary cell culture.

Our ultrastructural analysis confirmed that we isolated glioblastoma CSCs expressing the marker CD133. We observed that these cells possessed ultrastructural features similar to those of undifferentiated MSCs, such as spindle morphology with cytoplasmic extensions.

After extensive characterization of the CD133+ glioblastoma cells, we demonstrated that these cells recapitulated the phenotype of the original patient's tumor, after the cells were administered *in vivo* to non-immunocompromised animals. Histopathological examinations of the tumor revealed high cellularity of glial origin, nuclear atypia, vascular proliferation, invasiveness and a high Ki-67 proliferative index. These findings demonstrate that we were able to isolate stem cells responsible for tumorigenesis, thus demonstrating a correlation between the prognostic value and the expression of the stem cell marker CD133 in glioblastoma [31].

In this study, we showed that the CD133^+^ glioblastoma cells formed intracranial tumors in rats that were not immunocompromised, thus suggesting the involvement of local immunoresistance mechanisms or tumor-specific immunity avoidance through different mechanisms involving both the intrinsic properties of the glioma cells and microenvironmental factors [32, 33]. Notably, MSCs are able to avoid the immune response in allogenic and xenotransplants; however, the mechanism has not been fully elucidated but parallels tumor evasion. The MSC-like properties observed in the CD133^+^ cell population from this study may be important to sustain tumor growth and malignant progression, which is affected by the tumor microenvironment created by the non-neoplastic stroma composed of inflammatory and connective tissue components [34, 35, 36, 37, 38].

In summary, we showed that CD133^+^ glioblastoma-initiating cells express cell surface markers characteristic of MSCs. It is likely that these markers play a role in the biological and pathological behavior of the glioblastoma cells *in vivo* during malignant progression. These findings may interfere the development of effective treatment strategies that are commonly used for MSC-initiated tumors but are not currently considered for glioblastoma. Consequently, we suggest that the MSC-like properties of CD133^+^ glioblastoma-initiating cells confer pro-angiogenic, anti-apoptotic and immunomodulatory characteristics that may sustain tumor growth even in animals that are not immunocompromised. Therefore, tumor progression may be directed by reciprocal interaction between tumor stromal cells and tumor cells to create an appropriate environment for tumor aggressiveness. Future studies are necessary to elucidate the function of the MSC traits found in the CD133^+^ glioblastoma CSCs in the genesis, invasiveness and progression of glioblastomas.

## MATERIALS AND METHODS

In this study, we analyzed five samples of human primary glioblastomas obtained from adult patients undergoing resection at the Department of Neurology and Neurosurgery, Escola Paulista de Medicina - Universidade Federal de São Paulo (EPM-UNIFESP). All patients gave informed consent for participation in the study (UNIFESP - Ethical Committee).

### Establishment of the glioblastoma primary cell culture

Fresh glioblastoma samples were washed and minced in phosphate-buffered saline (PBS) (1X); this was followed by enzymatic dissociation with collagenase-I 0.3% (Sigma-Aldrich). The isolated cells were resuspended in Dulbecco's Modified Eagle's Medium-Low Glucose (DMEM-LG, Gibco/Invitrogen Corporation) supplemented with 200 mM of L-Glutamine, Antibiotic–Antimycotic (10,000 U/mL of sodium penicillin, 10,000 μg/mL of streptomycin sulfate and 25 μg/mL of amphotericin B; Thermo Fisher Scientific), and 10% Fetal Bovine Serum (Thermo Fisher Scientific). The cells were seeded in 25-cm^2^ culture flasks and maintained at 37°C with 5% CO_2_. The culture medium was changed every other day.

### Glioblastoma-derived neurosphere culture

The glioblastoma cells obtained in the primary culture described above were resuspended in “tumor brain stem cell medium” (Dulbecco's Modified Eagle's Medium/F12; Thermo Fisher Scientific), supplemented with N-2 (Thermo Fisher Scientific), epidermal growth factor (EGF, 20 ng/mL; Thermo Fisher Scientific), basic fibroblast growth factor (bFGF, 20 ng/mL; Thermo Fisher Scientific), leukemia inhibitory factor (LIF, 10 ng/μl; EMD Millipore) and B-27 (1:50; Thermo Fisher Scientific). Viable cells were seeded in 24-well plates at a density of 2×104 cells/cm^2^. The cells were maintained in a humidified incubator (Thermo Fisher Scientific, Waltham, MA) with 5% CO^2^ at 37*°C*, and the culture medium was changed every three days.

### Purification of the glioblastoma cells with CD133 microbeads and preparation of the tumor subspheres

The neurosphere colonies were dissociated using StemPro Accutase (Thermo Fisher Scientific) and maintained at room temperature for 10 min. The cells were labeled with CD133 magnetic microbeads (MACS; Miltenyi Biotec) and selected with an affinity column according to the manufacturer's instructions (Miltenyi Biotec). To verify the separation efficiency, the CD133+ cells were stained with CD133/2PE and evaluated by using flow cytometry (FACSAria, BD Biosciences, San Jose, CA) and analyzed with FACSDiva software (BD Biosciences, San Jose, CA) [39].

The CD133^+^ selected and CD133^−^ depleted cell populations were resuspended in Dulbecco's Modified Eagle's Medium/F12 and seeded in 24-well plates at a density of 1×10^3^ cells/cm^2^. Subsphere formation was observed in only the CD133^+^cells and was documented by using phase-contrast microscopy (Olympus IX51) [39].

### Immunophenotyping of the CD133^+^ glioblastoma cells by using flow cytometry

We analyzed the cell-surface expression of specific markers in the CD133^+^ cells subspheres by using commercially available monoclonal antibodies according to the manufacturer's instructions. Briefly, subspheres were harvested with StemPro Accutase Cell Dissociation Reagent (Thermo Fisher Scientific, Carlsbad, CA) and washed with PBS (pH=7.4). Next, the cells were stained with monoclonal antibodies and incubated in the dark for 30 min at room temperature. For intracellular staining, the cells were fixed (FACS Lysing Solution, BD Biosciences) and permeabilized (Permeabilization Solution 2, BD Biosciences, San Jose, CA). Human monoclonal antibodies against the following were used: CD133/2 PE (clone: 133/2; Miltenyi Biotec, Bergisch Gladbach, Germany), Nanog FITC, CD44 PE, Msh-1 PE, SSEA-3 FITC, CD90 APC and CD44 PerCP-Cy5.5 (all from BD Biosciences, San Diego, CA). We also used the related isotypes or fluorescence minus one (FMO) controls. The data were acquired with a FACSAria flow cytometer (BD Biosciences, San Jose, CA) and analyzed by using FACSDiva (BD Biosciences, San Jose, CA) or FlowJo software (Tree Star, Ashland, OR).

The expression of the typical mesenchymal markers in the glioblastoma CD133^+^ cells were analyzed by using CD29 PE, CD44 PE, CD73 PE, CD90 APC, CD105 PE and CD166 PE antibodies from BD Biosciences (San Diego, CA) using methods previously described in Sibov and colleagues [40].

### Adipogenic differentiation capacity of the CD133+ glioblastoma cells

CD133^+^ glioblastoma cells were subjected to adipogenic differentiation *in vitro* according to previously described methods by Sibov and colleagues [25]. Briefly, the cells were plated at a density of 103 cells/cm^2^ in a 6-well culture plate. When the cells reached approximately 80% confluence, adipogenic induction medium was added and changed every other day until 21 d. The adipogenic culture medium contained: 10 μg/mL of insulin (Sigma-Aldrich), 100 μM of indomethacin (Sigma-Aldrich), 1μM of dexamethasone (Sigma-Aldrich), and 100 μg/mL of 3-isobutyl-1-methyl-xanthine (Sigma-Aldrich) in Minimum Essential Medium Alpha Medium (Thermo Fisher Scientific) with 10% fetal bovine serum. After 21 d, the cells were fixed with 4% paraformaldehyde and stained with 0.3% Oil Red O stain (Sigma-Aldrich), according to methods previously described by Sibov and colleagues [41]. The morphology of the cells was assessed with an IX51 inverted microscope (Olympus, Tokyo, Japan).

### CD133+ glioblastoma cell labeling with multimodal iron oxide nanoparticles conjugated to Rhodamine-B (MION-Rh)

Approximately 103 CD133^+^ glioblastoma cells were plated in 24-well plates. The cells were incubated overnight (for approximately 18 h at 37°C with 5% CO^2^) in Dulbecco's Modified Eagle's Medium/F12 and 40 μg Fe/mL MION-Rh. After incubation, the culture medium solution was removed, and the cells were washed twice with PBS (1X) to remove the extracellular MION-Rh. The labeled cells were treated with 0.25% TrypLE Express and then harvested and manually counted using 0.4% Trypan Blue staining (Thermo Fisher Scientific).

### Intracellular detection of MION-Rh in the labeled CD133+ glioblastoma cells

The CD133^+^ glioblastoma cells were washed twice with PBS (1X) and fixed with 4% paraformaldehyde. The cell nuclei were labeled with diamidino-2-phenylindole (DAPI, Sigma-Aldrich) and analyzed with an IX51 fluorescence microscope (Olympus, Tokyo, Japan) with a Rh-B filter (530 nm and 550 nm) to detect the MION-Rh

### Isolation and culture of umbilical cord-derived MSCs (UC-MSC)

Five umbilical cord and umbilical cord blood samples were collected with the informed consent of the donor's mother, with protocol approval from the ethics committee for research at the HSP, UNIFESP, São Paulo, Brazil. The samples were processed and cultured for 21 d, with a medium change every other day, according to previously described methods by Sibov and colleagues [40]. After 3 weeks, UC-MSCs with fibroblast morphology were the dominant cells in the culture. UC-MSCs were differentiated into three mesodermal lineages and characterized by using flow cytometry. All experiments were performed with cells in the fourth passage.

### Selection of CD133^+^ hematopoietic stem cells from human umbilical cord blood (UCB)

UCB was collected by puncturing the umbilical cord vein at the moment of birth (after the cord was cut) using sterile equipment and 250 mL blood bags containing 2 mL of anticoagulant. The volume of the collected blood ranged between 70 and 120 mL. A sample of 80 μL of blood was separated for further cell count. The blood was then diluted (1:2) in RPMI culture medium (Thermo Fisher Scientific), and the lymphomononuclear cells were separated by using Ficoll-Paque^TM^ Plus (GE Healthcare) at a 1:3 density gradient (Lehner; Holter, 2002). The cells were centrifuged at 400 xg for 35 min, and the fraction containing the lymphomononuclear cells was isolated using a 10 mL pipette and then washed in RPMI medium. The CD133^+^ cell population was purified by using MiniMACS microbeads affinity chromatography using anti-CD133 bound to magnetic beads (Miltenyi Biotec). The cells were filtrated with a 30 μm nylon filter, and the number of cells was determined by the cell count in an automatic counter (Coulter). The cells were centrifuged (400 xg for 5 min) and resuspended in 300 μL of a PBS solution containing 2 mM of EDTA and 0.5% BSA. For each 108 cells, 100 μL of FcR blocker and 100 μL of magnetic micro-spheres with CD133^+^ antibodies (6°C) were added. The labeled cells were separated on a chromatography column to isolate the CD133+ cells according to previously described methods by Pavon and colleagues [42].

### Transmission electron microscopy (TEM) of the CD133+ glioblastoma cells, CD133+ hematopoietic stem cells (UCBs) and the UC-MSCs

The cells were fixed in 1% glutaraldehyde and 0.2 M of cacodylate buffer for 2 h at 4°C, according to previously described methods for TEM by Pavon and colleagues [42].

### Stereotaxic implantation of the CD133+ glioblastoma cells labeled with MION-Rh

The present work was conducted according to the regulations of the Ethics in Animal Research Committee of the UNIFESP. The animals (n=5; male Wistar rats) were anesthetized with ketamine (55 mg/kg) and xylazine (11 mg/kg). The hair was then removed from the top of the head, and the head was fixed in the stereotaxic apparatus (Stoelting®, model 51700). A skin incision was made on the dorsal region of the skull, the periosteum was removed and a trepanation of the bone cap was made using a dental drill. The implantation position was determined and marked in the bone according to Swanson's stereotaxic guidelines at the following coordinates: 6.0 mm anteroposterior, 4.5 mm mediolateral and a depth of 1.8 mm. A Hamilton syringe was used to implant 104 CD133+ glioblastoma cells resuspended in10 μL of culture medium into the right caudate putamen (CPu). The cells were slowly injected over a 10 min period. To avoid drawing the injected solution back into the needle, the syringe was kept in position for an additional 2 min before slowly being withdrawn from the brain. The bone was then reassembled using bone wax, and the skin was sutured using cotton thread.

### Analysis of tumor development by using *in vivo* imaging and histopathological analysis

The FX PRO imaging system was used to obtain X-ray and fluorescence images. The animals were placed in dorsal recumbency and remained anesthetized with inhalational anesthetic consisting of 2% isoflurane with oxygen (2 L/min) during the image acquisition. We first acquired X-ray images of the skull. Next, fluorescence images of the MION-Rh labeled cells were obtained at an excitation of 540 nm and an emission of 585 nm, and the images were analyzed using multiplex-located software.

We performed immunohistochemical staining for glial fibrillary acidic protein (GFAP) and the Ki67 proliferation marker, and Prussian blue staining for MION-Rh.

## References

[1] Altaner C (2008). Glioblastoma and stem cell. Neoplasma.

[2] Altaner C (2012). Glioma cancer stem cells and their role in therapy. Atlas Genet. Cytogenet. Oncol. Haematol.

[3] Singh SK, Clarke ID, Terasaki M, Bonn VE, Hawkins C, Squire J, Dirks PB (2003). Identification of a cancer stem cell in human brain tumors. Cancer Res.

[4] Soltysova A, Altanerova V, Altaner C (2005). Cancer stem cells. Neoplasma.

[5] Zeppernick F, Ahmadi R, Campos B, Dictus C, Helmke BM, Becker N, Lichter P, Unterberg A, Radlwimmer B, Herold-Mende CC (2008). Stem cell marker CD133 affects clinical outcome in glioma patients. Clinical Cancer Research.

[6] Park DM, Rich JN (2009). Biology of Glioma Cancer Stem Cells. Mol. Cells.

[7] Dirks PB (2010). Brain tumor stem cells: the cancer stem cell hypothesis writ large. Mol. Oncol.

[8] Choy W, Nagasawa DT, Trang A, Thill K, Spasic M, Yang I (2012). CD133 as a Marker for Regulation and Potential for Targeted Therapies in Glioblastoma Multiforme. Neurosurg. Clin. N. Am.

[9] Brescia P, Richichi C, Pelicci G (2012). Current Strategies for Identification of Glioma Stem Cells: Adequate or Unsatisfactory?. Journal of Oncology.

[10] Li Zhong (2013). CD133: a stem cell biomarker and beyond. Exp. Hematol. Oncol.

[11] Vora P, Venugopal C, McFarlane N, Singh SK (2015). Culture and Isolation of Brain Tumor Initiating Cells. Curr Protoc Stem Cell Biol.

[12] Soeda A, Hara A, Kunisada T, Yoshimura S, Iwama T, Park DM (2015). The evidence of glioblastoma heterogeneity. Sci Rep.

[13] Lottaz C, Beier D, Meyer K, Kumar P, Hermann A, Schwarz J, Junker M, Oefner PJ, Bogdahn U, Wischhusen J, Spang R, Storch A, Beier CP (2010). Transcriptional profiles of CD133^+^ and CD133^−^ glioblastoma-derived cancer stem cell lines suggest different cells of origin. Cancer Res.

[14] Kappadakunnel M, Eskin A, Dong J, Nelson SF, Mischel PS, Liau LM, Ngheimphu P, Lai A, Cloughesy TF, Goldin J, Pope WB (2010). Stem cell associated gene expression in glioblastoma multiforme: relationship to survival and the subventricular zone. J. Neurooncol.

[15] Liu A, Yu X, Liu S (2013). Pluripotency transcription factors and cancer stem cells: small genes make a big difference. Chin. J. Cancer.

[16] Toda M, Iizuka Y, Yu W, Imai T, Ikeda E, Yoshida K, Kawase T, Kawakami Y, Okano H, Uyemura K (2001). Expression of the neural RNA-binding protein Musashi1 in human gliomas. Glia.

[17] Hemmati HD, Nakano I, Lazareff JA, Masterman-Smith M, Geschwind DH, Bronner-Fraser M, Kornblum HI (2003). Cancerous stem cells can arise from pediatric brain tumors. Proc. Natl. Acad. Sci. USA.

[18] Son MJ, Woolard K, Nam DH, Lee J, Fine HA (2009). SSEA-1 is an enrichment marker for tumor-initiating cells in human glioblastoma. Cell Stem Cell.

[19] Son MJ, Woolard K, Nam DH, Lee J, Fine HA (2009). SSEA-1 is an enrichment marker for tumor-initiating cells in human glioblastoma. Cell Stem Cell.

[20] Ricci-Vitiani L, Pallini R, Larocca LM, Lombardi DG, Signore M, Pierconti F, Petrucci G, Montano N, Maira G, De Maria R (2008). Mesenchymal differentiation of glioblastoma stem cells. Cell Death Differ.

[21] Giordano A, Galderisi U, Marino IR (2007). From the laboratory bench to the patient's bedside: an update on clinical trials with mesenchymal stem cells. J. Cell Physiol.

[22] Normanton M, Alvarenga HG, Hamerschlak N, Ribeiro A, Kondo A, Rizzo LV, Marti LC (2014). Interleukin 7 Plays a Role in T Lymphocyte Apoptosis Inhibition driven by mesenchymal stem cell without favoring proliferation and cytokines secretion. Plos. One.

[23] Tso CL, Shintaku P, Chen J, Liu Q, Liu J, Chen Z, Yoshimoto K, Mischel PS, Cloughesy TF, Liau LM, Nelson SF (2006). Primary glioblastomas express mesenchymal stem-like properties. Mol. Cancer Res.

[24] Angel Alvarez, Monowar Hossain, Elise Dantuma, Stephanie Merchant, Kiminobu Sugaya (2010). Nanog Overexpression Allows Human Mesenchymal Stem Cells to Differentiate into Neural Cells-Nanog Transdifferentiates Mesenchymal Stem Cells. Neuroscience & Medicine.

[25] Kucerova L, Matuskova M, Hlubinova K, Altanerova V, Altaner C (2010). Tumor cell behaviour modulation by mesenchymal stromal cells. Mol Cancer.

[26] Hoelzinger DB, Demuth T, Berens ME (2007). Autocrine factors that sustain glioma invasion and paracrine biology in the brain microenvironment. J. Natl. Cancer Inst.

[27] Oka N, Soeda A, Inagaki A, Onodera M, Maruyama H, Hara A, Kunisada T, Mori H, Iwama T (2007). VEGF promotes tumorigenesis and angiogenesis of human glioblastoma stem cells. Biochem. Biophys. Res. Commun.

[28] Donnenberg VS, Donnenberg AD, Zimmerlin L, Landreneau RJ, Bhargava R, Wetzel RA, Basse P, Brufsky AM (2010). Localization of CD44 and CD90 positive cells to the invasive front of breast tumors. Cytometry B. Clin. Cytom.

[29] He J, Liu Y, Zhu T, Dimeco F, Vescovi AL, Heth JA, Muraszko KM, Fan X, Lubman DM (2012). CD90 is identified as a candidate marker for cancer stem cells in primary high-grade gliomas using tissue microarrays.

[30] Bahnassy AA, Zekri AR, El-Bastawisy A, Fawzy A, Shetta M, Hussein N, Omran D, Ahmed AA, El-Labbody SS (2014). Circulating tumor and cancer stem cells in hepatitis C virus-associated liver disease. World J. Gastroenterol.

[31] Shin JH, Lee YS, Hong YK, Kang CS (2013). Correlation between the prognostic value and the expression of the stem cell marker CD133 and isocitrate dehydrogenase1 in glioblastomas. J. Neurooncol.

[32] Okada H, Kohanbash G, Zhu X, Kastenhuber ER, Hoji A, Ueda R, Fujita M (2009). Immunotherapeutic approaches for glioma. Critical Reviews Immunology.

[33] Albesiano E, Han JE, Lim M (2010). Mechanisms of local immunoresistance in glioma. Neurosurgery Clinics of North America.

[34] Valtieri M, Sorrentino AJ (2008). The mesenchymal stromal cell contribution to homeostasis. Cell Physiol.

[35] Li H, Fan X, Houghton J (2007). Tumor microenvironment: the role of the tumor stroma in cancer. J. Cell Biochem.

[36] Joyce JA, Pollard JW (2009). Microenvironmental regulation of metastasis. Nat. Rev. Cancer.

[37] Hu M, Polyak K (2008). Molecular characterisation of the tumor microenvironment in breast cancer. Eur. J. Cancer.

[38] Bergfeld SA, DeClerck Y.A (2010). Bone marrow-derived mesenchymal stem cells and the tumor microenvironment. Cancer Metastasis Rev.

[39] Pavon LF, Marti LC, Sibov TT, Malheiros SM, Brandt RA, Cavalheiro S, Gamarra LF (2014). In vitro Analysis of Neurospheres Derived from Glioblastoma Primary Culture: A Novel Methodology Paradigm. Front. Neurol.

[40] Sibov TT, Pavon LF, Oliveira DM, Marti LC, Guilhen DD, Amaro E, Gamarra LF (2010). Characterization of adherent umbilical cord blood stromal cells regarding passage, cell number, and nano-biomarking utilization. Cell Reprogram.

[41] Sibov TT, Severino P, Marti LC, Pavon LF, Oliveira DM, Tobo PR, Campos AH, Paes AT, Amaro E, F Gamarra L, Moreira-Filho CA (2012). Mesenchymal stem cells from umbilical cord blood: parameters for isolation, characterization and adipogenic differentiation. Cytotechnology.

[42] Pavon LF, Gamarra LF, Marti LC, Amaro Junior E, Moreira-Filho CA, Camargo-Mathias MI, Okamoto OK (2008). Ultrastructural characterization of CD133^+^ stem cells bound to superparamagnetic nanoparticles: possible biotechnological applications. Journal of Microscopy.

